# Understanding U.S. Healthcare Providers’ Practices and Experiences with Molluscum Contagiosum

**DOI:** 10.1371/journal.pone.0076948

**Published:** 2013-10-14

**Authors:** Christine M. Hughes, Inger K. Damon, Mary G. Reynolds

**Affiliations:** Centers for Disease Control and Prevention, Division of High-Consequence Pathogens and Pathology, Poxvirus and Rabies Branch, Atlanta, Georgia, United States of America; Midwestern University, United States of America

## Abstract

**Introduction:**

Molluscum contagiosum is a common superficial skin infection caused by the poxvirus, Molluscum Contagiosum virus. The study objective is to obtain a better understanding of physician practices and experiences with molluscum contagiosum in order to focus informational and guidance material.

**Methods:**

A cross-sectional survey to assess medical practitioners’ knowledge and practices with molluscum contagiosum was conducted using the 2009 DocStyles survey. Questions regarding category and number of molluscum contagiosum patients seen, treatments used and advice given to patients were included in the survey.

**Results:**

Dermatologists saw the most cases, with the majority seeing 51–100 molluscum contagiosum cases/year. The most common cases seen were children with multiple lesions and adults with genital lesions. Respondents were most likely to recommend treatment to immunocompromised individuals, HIV patients, adults with genital lesions and children with multiple lesions. Cryotherapy was the top choice for all specialties with the exception of OB/GYNs, whose top choice was curettage. “Avoid intimate contact until lesions resolve”, “Avoid touching lesions to reduce further spread”, and “Don’t be concerned, this will go away” were the top advice choices.

**Discussion:**

Most survey respondents have dealt with molluscum contagiosum in their practice during the previous year. Overall, respondents picked appropriate choices for treatment and advice given; however some ineffective or unnecessary treatments were chosen and recommendations to prevent spread were chosen infrequently. Knowledge gaps for appropriate transmission precaution advice might cause unnecessary spread or autoinoculation. This survey has demonstrated that molluscum contagiosum is a common infection seen by many types of practitioners and therefore guidance on treatment considerations and infection control is valuable.

## Introduction

Molluscum contagiosum (MC) is a common superficial skin infection caused by the poxvirus, Molluscum Contagiosum virus (MCV). MC is characterized by painless white or skin-colored papular skin-lesions that are generally 2 to 5 mm in size, though early lesions can be smaller. MC patients typically have 1–20 lesions, although even healthy patients may have a hundred or more lesions. MC, in normal hosts, is benign, limited to the skin, but lesions can take as long as 6–12 months to resolve [1]. Immunocompromised patients can develop very large (>15 mm) and numerous lesions, and bacterial superinfections [2]–[4]. There has also been a report of molluscum contagiosum viral DNA in the blood of severely immunocompromised individuals, indicating possible viremia [5]. Children with atopic dermatitis may have higher numbers of MC lesions and an increased likelihood of molluscum dermatitis [6]. Children tend to have lesions on their trunk, limbs, or face [6]–[8]. In adults, lesions generally occur on the genitals, inner thighs and abdomen as transmission is associated with close sexual contact [9]–[11].

MCV is spread via direct human-to-human contact and via fomites [8], [12]–[16]. Autoinoculation can occur resulting in spread to other areas of the body. Covering lesions with clothing and/or bandages is one effective way to prevent spread. Good hand hygiene, avoiding touching the lesions and disinfection of potential fomites are also useful. For example, an individual with MC lesions may be advised to avoid sharing towels with other persons, in order to minimize risks for transmission. Patients who do not receive appropriate information about transmission risks may inadvertently spread the infection to other parts of their body (autoinoculate) or to others with whom they have contact (in the household, at school, etc.). On the other hand, advice to restrict children with MC from daycare or school until the infection clears is not practical or necessary.

Treatment is not necessary for most cases of MC as lesions are generally self-limiting and resolve without scarring [1], [17]. MC can be treated with physical destruction of lesions by cryotherapy, curettage, laser, salicylic acid, and other methods; however these procedures can be painful and can themselves engender scarring [18]–[21]. Alternatively, immune modulating therapies such as topical imiquimod or cimetidine have been used in attempts to speed healing of MC lesions, however there is limited information to show these are effective and imiquimod has known side-effects [17], [22]–[24]. The lack of an effective treatment that can be administered without serious side effects, pain, and/or scarring leads to the prolongation of infection and increased opportunities for virus to spread. This constitutes yet another potential missed opportunity to stop transmission in the household and community.

In an effort to obtain a better understanding of physician practices and experiences regarding MC, a cross-sectional survey was conducted using DocStyles, a web-based survey of primary care physicians and other specialties. Little research has been done on the types of MC patients physicians are likely to treat, the advice given, and treatment used. If appropriate transmission precaution advice were available to health care providers and their patients, additional spread and the costs associated with excess medical visits could be avoided. In addition, practical advice given to the families and schools of children with MC can help prevent needless exclusion from school or daycare. Restricting attendance can cause numerous problems for patients’ families as parents struggle to stay home from work or find alternative child care. Achieving a reduction in the use of unnecessary or ineffective treatments can help prevent avoidable pain and scarring in MC patients. Understanding physicians’ knowledge and familiarity with MC will help us focus informational materials, such as websites and pamphlets.

## Methods

A cross-sectional design was used to survey physicians and other medical practitioners on current knowledge and practices regarding MC. The 2009 DocStyles survey was used for this study. DocStyles is a yearly survey of physicians and other specialties conducted by the public relations firm, Porter Novelli (www.porternovelli.com). The physician sample was drawn from Epocrates Honors Panel; an opt-in, verified panel of over 156,000 medical practitioners. Survey quotas were set to reach 1,000 primary care physicians, 250 pediatricians, 250 OB/GYNs, 250 dermatologists, 250 nurse practitioners, and 150 registered dietitians. Verification was achieved by checking each physician’s first name, last name, date of birth, medical school, and graduation date against the American Medical Association’s master file at the time of panel registration. Epocrates randomly selected a sample of eligible physicians from their main database to load into their invitation database. This sample was drawn to match AMA master file proportions for age, gender, and region. The registered dietitian and nurse practitioner samples were drawn from Epocrates’ Allied Health Panel of 473,000 health professionals, which includes 44,523 nurse practitioners and 1,587 dietitians. Responses from registered dietitians were not used for this study. All invitations included a link to the Web-based survey, which was hosted by ResearchNow. Respondents were paid an honorarium of $55–$95 for completing the survey.

The 2009 DocStyles survey included five multi-part questions related to experience and practice for MC patients. Potential respondents were asked what advice they had given to healthy MC patients in the past. They were provided with multiple choices and asked to select all that apply. They were also asked about the category of patients for whom they would recommend treatment as well as the type of treatment they would recommend. Potential respondents were again provided with multiple choices and asked to select all that apply. Respondents were then asked to give the number of MC cases they had seen in their practice during the last one year. Choices were 0,1–10,11–50,51–100,101–500, more than 500 cases. Finally, respondents were queried as to the nature of molluscum cases that they had seen during the last year (e.g., children with single lesion, children with multiple lesions, genital molluscum (adult), etc.). All questions were in a multiple-choice format with no option for free-text answers. In addition, personal characteristics such as sex, age, race/ethnicity, and professional characteristics, including type of practice and number of patients seen per week, were provided.

Basic frequencies were calculated for the types of MC cases seen during the last one year. Frequencies for the number of MC cases seen in the last one year and for the types of MC cases recommended for treatment were stratified by health care provider specialty. Logistic regression modeling was used to estimate the adjusted odds ratios (aORs) and 95% confidence intervals (CIs) for the types of treatment used and the advice given to MC patients adjusting for several variables. Physician characteristic variables adjusted for in this analysis include age (<40 (reference), 40–49, >50), sex, race (categorized into White (reference), African American, Asian, Other), specialty (Family/General Practitioner (reference), Internist, Pediatrician, OB/GYN, Dermatologist, Nurse Practitioner), number of patients seen per week (≤50 (reference),51–100,101–150, >150), and type of practice (Individual Practice (reference), Group Practice, Hospital or Clinic). Data analysis was performed using SAS v9.3.

## Results

**Figure 1:**
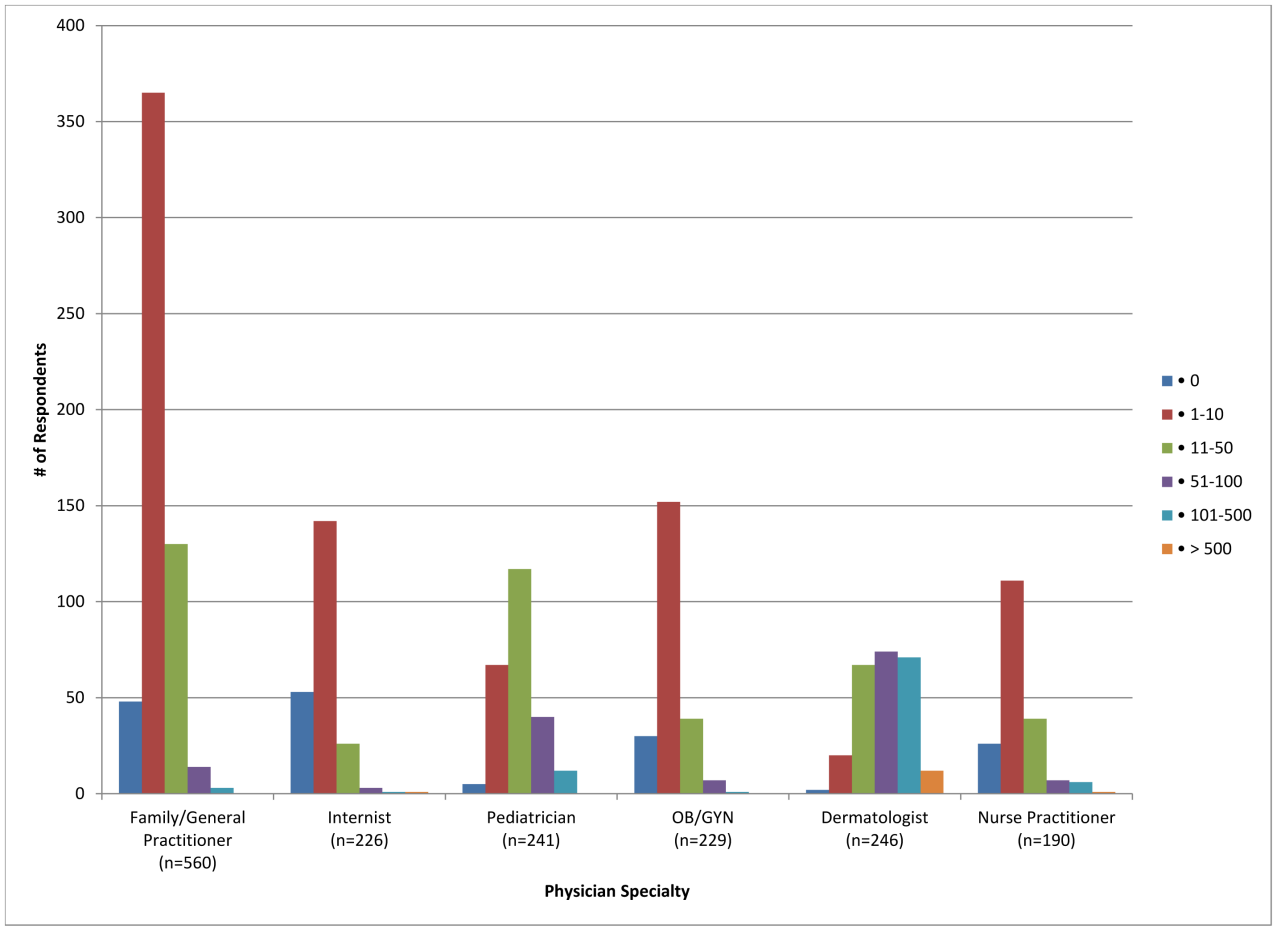
Number of molluscum contagiosum cases seen in the last one year for each specialty.

**Figure 2:**
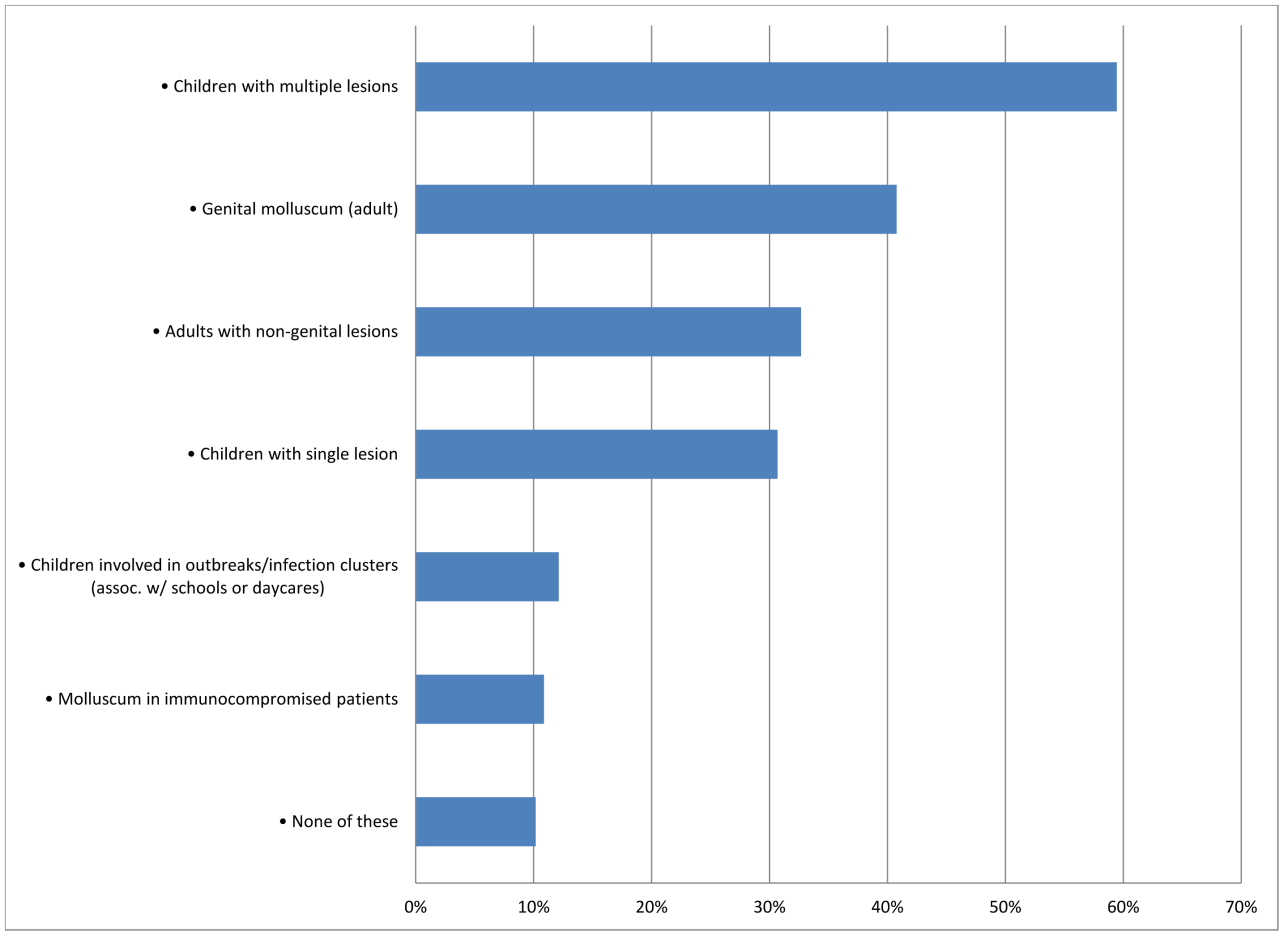
Percent respondents who reported seeing these types of molluscum contagiosum cases in the last one year.

A total of 2000 respondents were included in the survey, excluding registered dieticians. Those who answered “Not applicable to my practice” when asked about advice given to MC patients were also excluded (n = 308). The majority of respondents were family/general practitioners (30.45%), white (76.9%), male (61%), and work in a group practice (63.55%) (Table 1). Of these, most reported having seen between 1 and 10 cases of molluscum in the last year (50.65%); 48.55% of pediatricians saw 11–50 cases in the last year (Figure 1). Dermatologists reported seeing the most cases, with 30.08% seeing 51–100 cases in the last year. Twelve dermatologists (4.88%) reported seeing over 500 cases. The most common types of molluscum cases seen by respondents in the last one year were children with multiple lesions, followed by genital molluscum in adults (Figure 2). Pediatricians and dermatologists were the most likely to report having seen a child with multiple molluscum lesions (95.4% and 94.7% respectively). Dermatologists and OB/GYNs were most likely to report seeing a case of adult genital molluscum (84.5% and 80.8% respectively). The least common type of case seen was in immunocompromised patients, with dermatologists being the most likely to have seen at least one such patient during the previous year (37.8%).

**Table 1:** Demographic and other characteristics of DocStyles respondents, 2009.

Demographic characteristics	n	%
Total	2000	100.00%
**Age (in years)**		
• <40	706	35.30%
• 40–49	636	31.80%
• >50	658	32.90%
**Sex**		
• Male	1220	61.00%
• Female	780	39.00%
**Race**		
• White	1538	76.90%
• African American	77	3.85%
• Asian	289	14.45%
• Other	96	4.80%
**Ethnicity**		
• Hispanic	98	4.90%
• Non-Hispanic	1902	95.10%
**Specialty**		
• Family/General Practitioner	609	30.45%
• Internist	391	19.55%
• Pediatrician	250	12.50%
• OB/GYN	250	12.50%
• Dermatologist	250	12.50%
• Nurse Practitioner	250	12.50%
**Number of patients seen** **per week**		
• ≤50	192	9.60%
• 51–100	912	45.60%
• 101–150	641	32.05%
• >150	244	12.75%
**Years in Practice**		
• ≤10 years	856	42.80%
• 11–20 years	661	33.05%
• >20 years	483	24.15%

Overall, respondents were most likely to recommend treatment for molluscum cases in immunocompromised and HIV patients followed by adults with genital lesions and children with multiple lesions in one area (Table 2). Respondents were least likely to recommend treatment for adults with one non-genital lesion. A large majority of dermatologists responded that they would treat every type of patient, with only 1.22% responding that they would not treat any type of patient listed (Table 2). Pediatricians were the most likely to respond that they would not treat any type of patient. Pediatricians were the least likely to indicate that being enrolled in school or daycare was reason enough to recommend treatment to children.

**Table 2:** Frequency of responses for the question “Following a diagnosis of molluscum, which of the following types of patients would you likely recommend some form of treatment for the infection (select all that apply).”

		Specialty					
	All	Family/General Practitioner	Internist	Pediatrician	OB/GYN	Dermatologist	Nurse Practitioner
Response	% (n)	% (n)	% (n)	% (n)	% (n)	% (n)	% (n)
Child w/one lesion or widely scattered lesions	38.10% (644)	38.21% (214)	28.76% (65)	24.07% (58)	21.40% (49)	81.71% (201)	30.00% (57)
Otherwise healthy adult w/genital molluscum	49.65% (840)	47.86% (268)	44.25% (100)	18.26% (44)	56.77% (130)	88.62% (218)	42.11% (80)
Otherwise healthy adult w/one lesion (not on genitals)	23.23% (393)	19.46% (109)	14.16% (32)	4.15% (10)	20.96% (48)	71.54% (176)	9.47% (18)
Otherwise healthy adult w/multiple lesions(not on genitals)	41.67% (705)	40.36% (226)	36.28% (82)	14.94% (36)	41.48% (95)	84.55% (208)	30.53% (58)
Child w/multiple lesions in one area	47.93% (811)	48.57% (272)	30.53% (69)	46.06% (111)	27.07% (62)	88.21% (217)	42.11% (80)
Child enrolled in daycare	34.04% (576)	30.89% (173)	27.88% (63)	18.67% (45)	19.21% (44)	78.46% (193)	30.53% (58)
Child enrolled in school	29.55% (500)	26.07% (146)	21.24% (48)	13.69% (33)	15.72% (36)	76.42% (188)	25.79% (49)
Patients with HIV	55.02% (931)	52.86% (296)	57.08% (129)	40.66% (98)	46.72% (107)	83.74% (206)	50.00% (95)
Immunocompromised patients(not people with HIV)	62.94% (1048)	58.21% (326)	65.93% (149)	58.51% (141)	51.97% (119)	82.52% (203)	57.89% (110)
None of these	11.29% (191)	10.71% (60)	8.85% (20)	17.84% (43)	15.28% (35)	1.22% (3)	15.79% (30)

Dermatologists were over 3 times more likely to discourage over-the-counter medications or homeopathic remedies (aOR = 3.57, CI = 1.79–7.11) and pediatricians were almost 5 times more likely to refer patients to a dermatologist (aOR = 4.95, CI = 3.56–6.90) compared to family/general practitioners (Table 3). Nurse practitioners were over two times more likely to say they never recommend treatment in an otherwise healthy patient compared to family/general practitioners (aOR = 2.39, CI = 1.24–4.60).

**Table 3:** Frequencies and adjusted odds ratios of various characteristics with treatment responses for respondents.

	I discourage use of homeopathic remedies or over-the-counter products†			Refer to dermatologist for consultation			I never recommend treatment in an otherwise healthy patient		
Characteristic	%	aOR*	95% CI	%	aOR*	95% CI	%	aOR*	95% CI
**Specialty**									
• Family/General Practitioner (reference)	4.11%	1	–	25.00%	1	–	4.82%	1	–
• Internist	4.42%	1.65	(0.75–3.63)	**43.36%**	**2.37**	**(1.68–3.33)**	7.52%	1.32	(0.68–2.55)
• Pediatrician	4.98%	1.43	(0.69–2.96)	**61.83%**	**4.95**	**(3.56–6.90)**	5.39%	0.97	(0.48–1.95)
• OB/GYN	3.49%	0.86	(0.38–1.98)	**17.03%**	**0.6**	**(0.40–0.89)**	7.42%	1.52	(0.80–2.89)
• Dermatologist	**8.94%**	**3.57**	**(1.79–7.11)**	**5.69%**	**0.19**	**(0.10–0.34)**	**0.41%**	**0.09**	**(0.01–0.64)**
• Nurse Practitioner	1.58%	0.69	(0.19–2.51)	34.74%	1.29	(0.87–1.92)	**13.16%**	**2.39**	**(1.24–4.60)**
**Age (in years)**									
• <40 (reference)	3.84%	1	–	26.04%	1	–	5.18%	1	–
• 40–49	3.67%	1.09	(0.58–2.08)	31.01%	1.11	(0.83–1.47)	6.06%	1.13	(0.67–1.92)
• >50	**6.39%**	**1.87**	**(1.03–3.41)**	33.03%	1.28	(0.95–1.72)	6.57%	1.32	(0.77–2.26)

* Adjusted for specialty, age, sex, race, work setting, number of patients seen per week.

† There are no known effective over-the-counter products or homeopathic remedies approved for the treatment of molluscum contagiosum.

Cryotherapy was the top treatment choice for all physician specialties with the exception of OB/GYNs, whose top choice was curettage (Table 4). Internists, pediatricians, OB/GYNs, and nurse practitioners were less likely to choose cyrotherapy as compared to family/general practitioners. Cryotherapy was more likely to be picked by females compared to males. Physicians older than 40 years of age were more likely to choose curettage than those under 40. Laser therapy was more likely to be picked by internists, while pediatricians and dermatologists were less likely to select this as a possible treatment option. OB/GYNs were less apt than family/general practitioners to choose salicylic acid or oral cimetidine. Salicylic acid was more likely to be picked as a treatment by practitioners who self-described as being Asians and ‘other race’ compared to those who self-described as being white. Dermatologists were over 4 times more likely than family/general practitioners to prescribe imiquimod (aOR = 4.81, CI = 3.38–6.83) while internists were the least likely to say they would select this treatment.

**Table 4:** Frequencies and adjusted odds ratios of various characteristics with treatment option responses for respondents.

	Cryotherapy			Curettage			Salicylic acid			Imiquimod		
Characteristic	%	aOR*	95% CI	%	aOR*	95% CI	%	AOR*	95% CI	%	aOR*	95% CI
**Specialty**												
• Family/GeneralPractitioner (reference)	75.71%	1	–	39.11%	1	–	25.00%	1	–	27.14%	1	–
• Internist	**53.98%**	**0.35**	**(0.25–0.50)**	**22.57%**	**0.49**	**(0.34–0.71)**	22.12%	0.73	(0.50–1.07)	**16.81%**	**0.59**	**(0.38–0.88)**
• Pediatrician	**43.15%**	**0.23**	**(0.17–0.32)**	**29.46%**	**0.68**	**(0.49–0.95)**	21.99%	0.82	(0.56–1.18)	20.75%	0.76	(0.52–1.09)
• OB/GYN	**44.10%**	**0.25**	**(0.18–0.35)**	**60.70%**	**2.41**	**(1.74–3.32)**	**15.72%**	**0.58**	**(0.38–0.87)**	30.57%	1.27	(0.90–1.79)
• Dermatologist	80.89%	1.44	(0.97–2.14)	**61.79%**	**3.31**	**(2.35–4.67)**	25.20%	0.91	(0.62–1.33)	**67.07%**	**4.81**	**(3.38–6.83)**
• Nurse Practitioner	**48.42%**	**0.37**	**(0.25–0.53)**	**22.63%**	**0.45**	**(0.30–0.68)**	15.79%	0.64	(0.40–1.04)	23.16%	0.95	(0.62–1.46)
**Age (in years)**												
• <40 (reference)	61.44%	1	–	33.56%	1	–	22.37%	1	–	36.23%	1	–
• 40–49	**66.24%**	**1.55**	**(1.19–2.03)**	**39.82%**	**1.52**	**(1.16–1.98)**	22.39%	1.04	(0.78–1.40)	29.36%	0.83	(0.63–1.09)
• >50	57.12%	1.01	(0.77–1.33)	**46.90%**	**1.95**	**(1.48–2.56)**	20.99%	0.98	(0.72–1.33)	**25.91%**	**0.69**	**(0.51–0.92)**

* Adjusted for specialty, age, sex, race, work setting, number of patients seen per week.

When asked about the advice they would give to a molluscum patient, “Avoid intimate contact until lesions resolve”, “Avoid touching lesions to reduce further spread” and “Don’t be concerned, this will go away” were the overall top picks (44.74%, 42.79%, and 41.61% respectively). Only 11.8% of all respondents selected “Cover lesion with bandage”. Pediatricians were more likely to choose “Don’t be concerned, this will go away” and less likely to choose “Avoid school until the lesions resolve”, “Wash hands frequently”, “Disinfect household surfaces”, and “Don’t let others touch patient’s towels and sheets” when compared to general practitioners (Table 5). Older physicians (>50 yrs.) were more likely to recommend “Scratch lesion to promote inflammation and healing” (aOR (CI) = 2.14, (1.10–4.17)), and less likely to recommend “Cover lesion with bandage or dressing“ (7.75%, aOR (CI) = 0.47 (0.32–0.69), “Wash hands frequently”, “Avoid touching lesions”, “Don’t let others touch patient’s towels and sheets”, and “Avoid swimming pools until lesions resolve” when compared to those less than 40 yrs. Dermatologists are more likely to recommend avoiding contact sports until lesions resolve than general practitioners (aOR = 2.59, CI = 1.85–3.64) (Table 5).

**Table 5:** Frequencies and adjusted odds ratios of various characteristics with responses for advice given to patients.

	Don’t be concerned, this will go away		Wash hands frequently		Avoid contact sports until lesions resolve		Avoid intimate contact until lesions resolve (adult)		Avoid touching lesion to reduce further spread		Avoid school until the lesions resolve (children)	
Characteristic	%	aOR* (95% CI)	%	aOR* (95% CI)	%	aOR* (95% CI)	%	aOR* (95% CI)	%	aOR* (95% CI)	%	aOR* (95% CI)
**Specialty**												
• Family/GeneralPractitioner (reference)	42.53%	1	29.23%	1	25.12%	1	35.63%	1	30.87%	1	4.11%	1
• Internist	**14.07%**	**0.22 (0.16–0.31)**	**22.51%**	**0.67 (0.50–0.92)**	**16.37%**	**0.54 (0.39–0.76)**	**26.60%**	**0.63 (0.47–0.85)**	**18.41%**	**0.48 (0.35–0.67)**	5.37%	0.95 (0.51–1.80)
• Pediatrician	**74.00%**	**4.13 (2.95–5.78)**	**15.60%**	**0.44 (0.30–0.65)**	**12.00%**	**0.38 (0.25–0.58)**	**9.60%**	**0.19 (0.21–0.30)**	**38.40%**	**1.4 (1.02–1.92)**	**1.60%**	**0.3 (0.10–0.90)**
• OB/GYN	**22.00%**	**0.37 (0.26–0.52)**	**44.80%**	**2.02 (1.48–2.75)**	**15.60%**	**0.56 (0.38–0.82)**	**58.00%**	**2.62 (1.93–3.56)**	**42.80%**	**1.71 (1.25–2.33)**	4.80%	1.25 (0.61–2.58)
• Dermatologist	**27.20%**	**0.53 (0.38–0.75)**	34.40%	1.05 (0.75–1.48)	**47.20%**	**2.59 (1.85–3.64)**	**70.80%**	**3.97 (2.82–5.58)**	**69.20%**	**4.19 (2.98–5.88)**	4.40%	0.74 (0.34–1.61)
• Nurse Practitioner	**32.80%**	**0.60 (0.42–0.84)**	36.80%	1.41 (0.99–1.99)	18.00%	0.67 (0.44–1.01)	36.00%	1.09 (0.77–1.54)	35.20%	1.05 (0.74–1.50)	2.40%	0.58 (0.22–1.52)
**Age (in years)**												
• <40 (reference)	64.73%	1	33.57%	1	21.95%	1	39.94%	1	43.91%	1	5.52%	1
• 40–49	62.89%	1.01 (0.78–1.30)	29.09%	0.78 (0.61–1.00)	**22.80%**	**1.34 (1.01–1.78)**	36.01%	0.95 (0.74–1.22)	**34.12%**	**0.71 (0.55–0.90)**	4.09%	0.79 (0.46–1.35)
• >50	66.72%	0.80 (0.611.03)	**26.29%**	**0.66 (0.51–0.86)**	**22.64%**	**1.44 (1.07–1.93)**	37.39%	1.03 (0.80–1.33)	**29.94%**	**0.6 (0.46–0.77)**	**2.13%**	**0.45 (0.23–0.89)**
**Race**												
• White (reference)	36.35%	1	30.17%	1	21.59%	1	38.10%	1	36.28%	1	2.93%	1
• African American	29.87%	0.67 (0.39–1.16)	29.87%	0.93 (0.55–1.57)	16.88%	0.82 (0.43–1.54)	28.57%	0.69 (0.40–1.19)	38.96%	1.14 (0.70–1.88)	**9.09%**	**2.48 (1.05–5.89)**
• Asian	30.10%	0.80 (0.58–1.09)	26.99%	0.93 (0.69–1.27)	**28.03%**	**1.59 (1.16–2.18)**	**40.14%**	**1.36 (1.01–1.83)**	35.64%	0.97 (0.72–1.31)	**7.27%**	**1.94 (1.09–3.46)**
• Other	36.46%	1.18 (0.72–1.94)	31.25%	1.09 (0.68–1.73)	23.96%	1.39 (0.83–2.33)	34.38%	0.94 (0.58–1.52)	34.38%	0.93 (0.58–1.50)	6.25%	1.76 (0.71–4.36)

* Adjusted for specialty, age, sex, race, work setting, number of patients seen per week.

• Scratch lesion to promote inflammation and healing (>50 yrs, 5.93%, aOR (CI) = 2.14 (1.10–4.17)).

• Cover lesion with bandage or dressing (>50 yrs, 7.75%, aOR (CI) = 0.47 (0.32–0.69)).

## Discussion

Molluscum contagiosum is a common skin infection. Most of the practitioners who participated in the survey had dealt with the condition in their practice during the previous year. The bulk of respondents in the survey reported having seen between 1 and 10 cases in the last year. Pediatricians and dermatologists in this survey reported the most experience with MC cases, having seen an average of 11–50 and 51–100 cases, respectively, during the year prior to the survey. The considerable number of cases seen by pediatricians in this survey is consistent with prior reports of elevated MC incidence rates in young children [25]–[27].

Overall, health care providers were most likely to recommend treatment for immunocompromised patients, patients with HIV, adults with genital molluscum, and children with multiple lesions in one area. They were less likely to treat children with one or widely scattered lesions, which coincides with current recommendations (16). In contrast, dermatologists reported a greater readiness to treat MC regardless of the age of the patient or concurrent medical disorders such as HIV or other immunocompromising conditions. A possible explanation for this finding could be that cases are usually referred to a dermatologist by their primary physician specifically for the purpose of treatment. It is also possible that patients who seek care from a dermatologist may be experiencing a less benign manifestation of infection that might require treatment, or they may have lesions that are visible or cause embarrassment. Another explanation might be that dermatologists see so many patients with extensive MC that many believe treatment is worthwhile, even when the patient has minimal lesions. Dermatologists are equipped to routinely remove these types of lesions and therefore may be more inclined to do so.

Cryotherapy and curettage are the top choices for treatment for all health care providers and have been shown to be somewhat effective. However, these techniques can cause scarring, hypo or hyper pigmentation, and can be painful, especially for children [17], [19]. Curettage generally requires topical anesthetic. Imiquimod is a popular off-label MC treatment choice of dermatologists, however recent studies have shown it to be ineffective for molluscum and it is currently not approved for treatment of MC in children [28]. In addition, there have been case reports of adverse events such as fever, arthralgia, headache, myalgia, lymphadenopathy, febrile seizures and hypopigmentation scars when treating with imiquimod [22]–[24]. When considering these adverse events, pain, scarring and questionable effectiveness, treatment of such a benign disease may be considered excessive in many cases. However, the consideration of whether or not to treat is likely to be very different for more complicated cases, such as immunocompromised patients. If an effective treatment were available for all MC patients, the cost both financially in terms of doctors’ visits and anxiety could be drastically reduced for both adult patients and child patients and their families.

Most health care providers picked appropriate choices for advice given to MC patients. The top advice from family/general practitioners and pediatricians of “Don’t be concerned, this will go away” is helpful to ease the patient and the patient’s family’s concern about this infection and possibly prevent unnecessary treatment. The top choice for internists, OB/GYNs and dermatologists is “Avoid intimate contact until lesions resolve”. This advice and another top pick, “Avoid touching lesion to reduce further spread”, are effective for preventing spread to others and autoinoculation. Some of the Centers for Diseases Control and Prevention’s (CDC) recommendations (Table 6) to prevent spread, such as “Wash hands frequently”, “Cover lesion with a bandage or dressing”, “Don’t let others touch patient’s towels/sheets” were chosen infrequently by most health care providers. Dermatologists were more likely to choose these types of recommendations, which could be due to more experience with MC patients compared to other health care providers.

**Table 6:** CDC molluscum contagiosum transmission precaution recommendations.

Do not touch, pick or scratch lesions
Practice good hand hygiene
Keep lesions clean and covered with clothing or bandage
Cover lesions with watertight bandages before participating in contact sports or sharing equipment (swimming pools)
Do not share towels, clothing, or other personal items
Do not shave or have electrolysis on areas with lesions
Avoid sexual activities if lesions are in the genital area until you see a health care provider
There should be no reason to keep a child with MC home from school or daycare

* Additional information located at: http://www.cdc.gov/ncidod/dvrd/molluscum/index.htm.

Physicians over the age of 50 were less likely to recommend effective advice such as “Cover lesion with a bandage or dressing”, “Wash hands frequently”, “Avoid touching lesion to reduce further spread”. They were also more likely to offer advice that may be considered ineffective or detrimental, such as “Scratch lesion to promote inflammation and healing”. While there have been claims of scratching as a potential for helping lesions resolve, it is not recommended as it can cause autoinoculation and spread to others. A common query to the CDC hotline made by patients’ families and schools is whether a child with MC should attend school (unpublished data). MC is benign and can take months to resolve, therefore excluding children from school is considered excessive and unnecessary. Very few health care providers picked “Avoid school until the lesions resolve (children)” for advice offered.

Knowledge gaps for appropriate transmission precaution advice might cause unnecessary further spread or autoinoculation. In addition, unnecessary exclusion of children with MC from school or daycare can cause many problems for families. If appropriate transmission advice was provided to these children’s families and schools, the stresses of parents taking off work and losing income or finding alternative child care could be prevented. While the appropriate advice was chosen more often than inappropriate advice, the percent respondents was low for most of the survey choices, including those which CDC recommends for transmission precautions (Table 6).

Overall, survey respondents were able to choose appropriate treatments and advice for MC patients. The survey revealed some potentially unnecessary or ineffective treatment choices for MC patients. MC, in a normal host, is benign and treatment is often unnecessary and can cause unwanted side effects. However the persistence of lesions may influence practitioners’ treatment plans. Respondents were generally able to provide appropriate hygiene advice to MC patients. Increased educational materials would be helpful to reinforce the recommendations to prevent spread to others and prevent autoinoculation. A better understanding of why non-standard advice is given will help to focus educational materials. Prevention of MC spread and autoinoculation can decrease anxiety and relieve burden of patients and physicians. Uniformity of recommendations and emphasis on low-cost, low-risk therapies and interventions would be beneficial as well. This survey has demonstrated that MC is a common skin infection seen by many types of health care providers and therefore guidance on treatment considerations and infection control is valuable.
